# The effect of metabolic syndrome on prognosis of diffuse large B-cell lymphoma

**DOI:** 10.1007/s12094-024-03438-z

**Published:** 2024-03-30

**Authors:** Wenjing Xiong, Liru Li, Xue Hui, Yue Liu, Hongbin Li, Yue Zhang, Shu Zhao

**Affiliations:** Department of Clinical Oncology, Harbin Medical University Cancer Hospital, 150 Haping Road, Harbin, 150040 China

**Keywords:** Diffuse large B-cell lymphoma, Metabolic syndrome, Overall survival, Progression-free survival

## Abstract

**Purpose:**

Metabolic syndrome (MetS), characterized by insulin resistance, is closely associated with the prognosis of various cancer types, but has not been reported in diffuse large B-cell lymphoma (DLBCL). The aim of this study is to examine how other clinicopathological variables and the MetS influence the prognosis of DLBCL.

**Methods:**

Clinical and pathological data were collected from 319 patients with DLBCL who were admitted to our hospital between January 2012 and December 2020. The data accessible with SPSS 27.0 enables the utilization of various statistical methods for clinical data analysis, including independent sample *t* test and univariate and multivariate COX regression.

**Results:**

The presence of MetS was linked to both overall survival (OS) and progression-free survival (PFS), in addition to other clinicopathological characteristics as age, IPI score, rituximab usage, and Ki-67 expression level. This link with OS and PFS indicated a poor prognosis, as shown by survival analysis. Subsequent univariate analysis identified IPI score, Ki-67 expression level, tumor staging, rituximab usage, lactate dehydrogenase expression level, and the presence or absence of MetS as factors linked with OS and PFS. Furthermore, multivariate Cox regression analysis confirmed the independent risk factor status of IPI score, Ki-67 expression level, rituximab usage, and the presence of MetS in evaluating the prognosis of patients with DLBCL.

**Conclusion:**

This study’s findings indicate that patients with pre-treatment MetS had a poor prognosis, with relatively shorter OS and PFS compared to those without pre-treatment MetS. Furthermore, the presence of MetS, IPI score, Ki-67 expression level, and rituximab usage were identified as independent risk factors significantly affecting the prognosis of DLBCL.

## Introduction

Diffuse large B-cell lymphoma (DLBCL) is the most common type of non-Hodgkin’s lymphoma (NHL), with recent data suggesting that it accounts for approximately 30% of all new NHL cases globally each year [1]. It is worth noting that up to 40% of DLBCL cases are diagnosed with an extranodal primary site, indicating that the disease can occur in any part of the body. However, intranodal organs such as the tonsils, thymus, and spleen are more susceptible to the disease than extranodal organs. Despite significant improvements in patients’ survival rates due to the R-CHOP regimen treatment, some patients still experience disease progression [2]. Given the significant challenges associated with prognosis and treatment, researchers are actively working to improve the therapeutic outcomes for DLBCL patients and refine the prognostic evaluation system. As a result, there is an urgent need to explore additional factors that affect the prognosis of DLBCL in order to generate innovative ideas for clinical diagnosis and treatment.

Metabolic syndrome (MetS), initially described by Reaven in 1988, is a collection of metabolic disorders characterized by insulin resistance. Its primary components including obesity, dyslipidemia, hyperglycemia, hyperinsulinemia, and hypertension [3]. The definition of MetS has been continually refined by various organizations, with the current definition issued by NCEP-ATP III being widely accepted [4]. Numerous studies have shown a higher prevalence of various tumor types in patients with MetS, such as breast, bowel, colon, esophageal, gastric, and pancreatic. For instance, Elizabeth M et al. found that high-density lipoprotein cholesterol (HDL-C) and abdominal obesity in postmenopausal women increased the risk of breast cancer [5], while Buono et al. suggested that MetS was associated with a poor prognosis in patients with early-stage breast cancer, even when patients did not fully meet the diagnostic criteria for MetS [6]. Additionally, individual components of MetS have been found to correlate with specific cancer types. For example, obese women with breast cancer have been shown to have poorer survival rates than normal-weight women [7], and type 2 diabetes has been identified as an independent prognostic factor associated with poor overall survival in patients with early-stage breast cancer in an Asian study [8]. Notably, no study has yet explored the potential correlation between MetS and the prognosis of patients with DLBCL. Therefore, further research is needed to investigate the impact of MetS on the prognosis of DLBCL, providing a significant opportunity to advance clinical diagnosis and treatment.

## Materials and methods

### Sources of clinical data

The clinicopathological data from 319 DLBCL patients treated at our hospital between January 2012 and December 2020 were collected. This included basic information, such as gender, age, height, weight, Eastern Cooperative Oncology Group (ECOG) score, Ann Arbor stage, pathological typing, Ki-67 expression level, presence of B symptoms, the grouping of the number of extra conjunctival organ involvement, International Prognostic Index (IPI) score, the presence of bone marrow involvement, and whether rituximab was applied. Additionally, we collected data on lactate dehydrogenase (LDH) expression level, β2-microglobulin (β2-MG), and other clinicopathological data of the patients before chemotherapy. All patients are subject to regular follow-up visits, which included an assessment of their current status, recurrence evaluation, and documentation of the time and cause of death if applicable. The follow-up date for all patients was set as September 1, 2023.

### Inclusion criteria

1. The first diagnosis of DLBCL;
2. Patients’ relevant clinical data were complete;
3. All patients’ initially received full-dose CHOP or CHOP-like chemotherapy, regardless of whether rituximab was included.

Patients who met the above criteria were included in this study.

### Exclusion criteria

1. Patients with concurrent other malignant tumors;
2. Patients with active infectious diseases;
3. Patients with severe respiratory, cardiovascular, and neurological diseases.

Those with one of the above conditions must be excluded.

### Definition of metabolic syndrome

According to the National Cholesterol Education Program Adult Treatment Panel III (NCEP-ATP III) definition, MetS is diagnosed when three or more of the following five risk factors are present:

1. Obesity (waist circumference > 88 cm), which was replaced by body mass index (BMI) ≥ 25 kg/m^2^ in this study, supported by research data [9];
2. Triglycerides (TG) ≥ 150 mg/dL (1.7 mmol/L);
3. High-density lipoprotein cholesterol (HDL-C) < 50 mg/dL (1.3 mmol/L);
4. Systolic blood pressure (SBP) ≥ 130 and/or diastolic blood pressure (DBP) ≥ 85 mmHg;
5. Fasting plasma glucose (FPG) ≥ 110 mg/dL (≥ 6.1 mmol/L).

### Data analysis

Statistical analyses were conducted using SPSS 27.0 software. To examine potential correlations among multiple factors in patients with pre-treatment combined MetS and pre-treatment uncomplicated MetS, one-way analyses with independent samples *t* tests were performed. Additionally, to assess the prognostic aspect of the patients, Kaplan–Meier survival curves were utilized to generate PFS- and OS-related survival plots. Furthermore, a one-way single-factor Cox analysis was employed to investigate the relationship between clinical and pathological indicators and prognosis. Finally, multifactorial Cox regression was applied to analyze the independent prognostic factors affecting PFS and OS. The significance level was set at *P* < 0.05, indicating statistical significance of the observed differences.

## Results

### Baseline characteristics of patients

The study collected 319 patients diagnosed with DLBCL based on specific inclusion and exclusion criteria. Follow-up was conducted until September 1, 2023, revealing 265 survivors (83.1%) and 54 deaths (16.9%) at the end of the observation period. The patients’ age ranged from 15 to 85 years, with a median age of 58 years, comprising 129 patients with pre-treatment comorbid MetS and 190 patients without MetS (Table 1). Comparatively, patients with pre-treatment combined MetS exhibited older age (*P* = 0.029), higher IPI score (*P* = 0.016), and higher Ki-67 expression level (*P* = 0.017). Furthermore, a correlation was observed between the application of rituximab and MetS (*P* = 0.014). Additionally, patients with comorbid MetS demonstrated elevated fasting glucose, total cholesterol (TC), TG, LDL-C, SBP, DBP, BMI, as well as lower HDL-C in comparison to patients without MetS, with most of these differences being statistically significant and aligned with the definition of MetS (Table 2).

**Table 1:** The Clinical Characteristics of DLBCL Patients

Characteristics	Number (*n* = 319)
Age(years)	
≤ 60	180 (56.4%)
> 60	139 (43.6%)
Gender	
Male	139 (43.6%)
Female	180 (56.4%)
Hans typing	
GCB	89 (28.9%)
Non-GCB	230 (32.1%)
Ann Arbor stage	
I–II	210 (65.8%)
III–IV	109 (34.2%)
B symptom	
Yes	62 (19.4%)
No	257 (80.6%)
IPI score	
≤ 2	254 (79.6%)
> 2	65 (20.4%)
Rituximab	
Yes	267 (83.7%)
No	52 (16.3%)
Extranodal site	
0–1	287 (90%)
≥ 2	32 (10%)
ECOG	
< 2	259 (81.2%)
≥ 2	60 (18.8%)
Ki-67(%)	
≤ 70	112 (35.1%)
> 70	207 (64.9%)
Bone marrow involvement	
Yes	5 (1.6%)
No	314 (98.4%)
MetS	
MetS	129 (40.4%)
Non-MetS	190 (59.6%)
LDH	
Normal	213 (66.8%)
High	106 (33.2%)

*GCB* germinal center B-cell, *non-GCB* non-germinal center B-cell, *IPI* international prognostic index, *ECOG* eastern cooperative oncology group, *MetS*, metabolic syndrome, *LDH* lactate dehydrogenase

**Table 2:** Relationship between clinicopathological characteristics and MetS status in 319 DLBCL patients

Characteristics	METs		χ2	*P*
	METs	Non-METs		
Age(years)				
≤ 60	63	117	5.073	0.029*
> 60	66	73		
Gender				
Male	60	79	0.76	0.421
Female	69	111		
Hans typing				
GCB	38	51	0.261	0.613
Non-GCB	91	139		
Ann Arbor stage				
I–II	83	127	0.214	0.718
III–IV	46	63		
B symptom				
Yes	28	34	0.713	0.471
No	101	156		
IPI score				
≤ 2	94	160	6.092	0.016*
> 2	35	30		
Rituximab				
Yes	116	151	6.148	0.014*
No	13	39		
Extranodal site				
0–1	117	170	0.128	0.85
≥ 2	12	20		
ECOG				
< 2	99	160	2.805	0.109
≥ 2	30	30		
Ki-67(%)				
≤ 70	35	77	6.051	0.017*
> 70	94	113		
Bone marrow involvement				
Yes	2	3	0	1
No	127	187		
LDH				
Normal	80	133	2.208	0.147
High	49	57		
β2-MG	2.264 (± 1.007)	2.183 (± 1.079)		0.497
FPG (mmol/L)	5.819 (± 1.511)	5.056 (± 0.757)		< 0.001*
TC (mmol/L)	4.806 (± 0.947)	4.725 (± 1.063)		0.484
TG (mmol/L)	2.115 (± 0.895)	1.414 (± 0.682)		< 0.001*
HDL-C (mmol/L)	1.034 (± 0.282)	1.187 (± 0.316)		< 0.001*
LDL-C (mmol/L)	3.392 (± 0.809)	3.306 (± 0.860)		0.371
SBP (mmHg)	138.101 (± 17.068)	120.753 (± 14.188)		< 0.001*
DBP (mmHg)	83.954 (± 10.222)	74.868 (± 9.111)		< 0.001*
Height (cm)	164.892 (± 7.472)	164.716 (± 7.534)		0.838
Weight (kg)	59.547 (± 9.252)	69.115 (± 11.505)		< 0.001*
BMI (kg/m^2^)	25.379 (± 3.589)	21.898 (± 2.739)		< 0.001*

*GCB* germinal center B-cell, *non-GCB* non-germinal center B-cell, *IPI* international prognostic index, *ECOG* eastern cooperative oncology group, *MetS* metabolic syndrome, *LDH* lactate dehydrogenase, *β2-MG*, β2-microglobulin, *FPG* fasting plasma glucose, *TC* total cholesterol, *TG* triglycerides, *HDL-C* high-density lipoprotein cholesterol, *LDL-C* low-density lipoprotein cholesterol, *SBP* systolic blood pressure, *DBP* diastolic blood pressure, *BMI* body mass index

*P < 0.05

### Unifactorial and multifactorial analyses of prognosis

In our univariate analysis, the results revealed significant associations between several factors and OS. Specifically, age (*P* = 0.006), Ki-67 expression level (*P* = 0.013), Ann Arbor staging (*P* = 0.027), International Prognostic Index (IPI) score (*P* < 0.001), ECOG score (*P* = 0.007), rituximab application (*P* = 0.044), LDH expression level (*P* = 0.038), and the presence of combined MetS (*P* = 0.015) were found to be statistically associated with OS (Table 3). Additionally, factors such as Ann Arbor staging (*P* = 0.03), Ki-67 expression level (*P* = 0.044), IPI score (*P* < 0.001), rituximab application (*P* = 0.014), concurrent MetS (*P* = 0.01), and LDH expression level (*P* = 0.015) were also found to be significantly correlated with PFS (Table 4).

**Table 3:** Univariate analysis of OS

Characteristics		OS		χ^2^	*P*
		Yes	No		
Age (years)	≤ 60	21	159	8.131	0.006*
	> 60	33	106		
Gender	Male	25	114	0.196	0.655
	Female	29	151		
Hans typing	GCB	10	79	2.844	0.099
	Non-GCB	44	186		
Ann Arbor stage	I–II	28	182	5.647	0.027*
	III–IV	26	83		
B symptom	Yes	10	52	0.035	1
	No	44	213		
IPI score	≤ 2	32	222	16.616	< 0.001*
	> 2	22	43		
Rituximab	Yes	40	227	4.414	0.044*
	No	14	38		
Extranodal site	0–1	46	241	1.648	0.214
	≥ 2	8	24		
ECOG	< 2	36	223	8.98	0.007*
	≥ 2	18	42		
Ki-67(%)	≤ 70	11	101	6.199	0.013*
	> 70	43	164		
Bone marrow involvement	Yes	2	3	1.923	0.2
	No	52	262		
MetS	MetS	30	99	6.167	0.015*
	non-MetS	24	166		
LDH	Normal	29	184	5.003	0.038*
	High	25	81		
β2-MG		2.417 (± 1.136)	2.174 (1.028)		0.121
FPG (mmol/L)		5.164 (± 1.138)	5.405 (± 1.190)		0.172
TC (mmol/L)		4.719 (± 0.858)	4.767 (± 1.047)		0.761
TG (mmol/L)		1.840 (± 0.980)	1.669 (± 0.816)		0.176
HDL-C (mmol/L)		1.058 (± 0.280)	1.139 (± 0.316)		0.084
LDL-C (mmol/L)		3.374 (± 0.650)	3.334 (± 0.874)		0.751
SBP (mmHg)		131.13 (± 19.473)	127.083 (± 17.148)		0.124
DBP (mmHg)		79.833 (± 12.083)	78.279 (± 10.217)		0.325
Height (cm)		164.963 (± 7.790)	164.751 (± 7.452)		0.850
Weight (kg)		62.667 (± 11.513)	63.569 (± 11.196)		0.591
BMI (kg/m^2^)		22.969 (± 3.571)	23.375 (± 3.543)		0.444

*GCB* germinal center B-cell, *non-GCB* non-germinal center B-cell, *IPI* international prognostic index, *ECOG* eastern cooperative oncology group, *MetS* metabolic syndrome, *LDH* lactate dehydrogenase, *β2-MG* β2-microglobulin, *FPG* fasting plasma glucose, *TC* total cholesterol, *TG* triglycerides, *HDL-C* high-density lipoprotein cholesterol, *LDL-C* low-density lipoprotein cholesterol *SBP* systolic blood pressure, *DBP* diastolic blood pressure, *BMI* body mass index

*P < 0.05

**Table 4:** Univariate analysis of PFS

Characteristics		PFS		χ^2^	*P*
		Yes	No		
Age(years)	≤ 60	48	132	4.206	0.051
	> 60	52	87		
Gender	Male	46	93	0.349	0.627
	Female	54	126		
Hans typing	GCB	26	63	0.261	0.687
	non-GCB	74	156		
Ann Arbor stage	I–II	57	153	5.05	0.03*
	III–IV	43	66		
B symptom	Yes	20	42	0.03	0.879
	No	80	177		
IPI score	≤ 2	67	187	14.307	< 0.001*
	> 2	33	32		
Rituximab	Yes	76	191	6.328	0.014*
	No	24	28		
Extranodal site	0–1	87	200	1.422	0.235
	≥ 2	13	19		
ECOG	< 2	75	184	3.656	0.064
	≥ 2	25	35		
Ki-67(%)	≤ 70	27	85	4.205	0.044
	> 70	73	134		
Bone marrow involvement	Yes	2	3	0.177	0.65
	No	98	216		
MetS	MetS	51	78	6.745	0.01*
	Non-MetS	49	141		
LDH	Normal	57	156	6.268	0.015*
	High	43	63		
β2-MG		2.279 (± 1.123)	2.186 (± 1.016)		0.463
FPG (mmol/L)		5.230 (± 1.007)	5.426 (± 1.226)		0.169
TC (mmol/L)		4.793 (± 0.915)	4.742 (± 1.062)		0.678
TG (mmol/L)		1.817 (± 0.977)	1.643 (± 0.777)		0.089
HDL-C (mmol/L)		1.100 (± 0.239)	1.137 (± 0.320)		0.322
LDL-C (mmol/L)		3.379 (± 0.711)	3.323 (± 0.893)		0.582
SBP (mmHg)		128.56 (± 18.523)	127.406 (± 17.188)		0.588
DBP (mmHg)		78.61 (± 11.478)	78.511(± 10.128)		0.938
Height (cm)		165.31 (± 7.935)	164.548(± 7.296)		0.401
Weight (kg)		63.845 (± 11.643)	63.221(± 11.068)		0.646
BMI (kg/m^2^)		23.298 (± 3.462)	23.309(± 3.591)		0.979

*GCB* germinal center B-cell, *non-GCB* non-germinal center B-cell, *IPI* international prognostic index, *ECOG* eastern cooperative oncology group, *MetS* metabolic syndrome, *LDH* lactate dehydrogenase, *β2-MG* β2-microglobulin, *FPG* fasting plasma glucose, *TC* total cholesterol, *TG* triglycerides, *HDL-C* high-density lipoprotein cholesterol, *LDL-C* low-density lipoprotein cholesterol, *SBP* systolic blood pressure, *DBP* diastolic blood pressure, *BMI* body mass index

*P < 0.05

To uncover additional independent clinical features related to prognosis, a multifactorial Cox regression analysis was conducted on various factors, including IPI score, clinical stage, Ki-67 expression level, rituximab application status, LDH expression level, and the presence of MetS. The analysis revealed that IPI score, Ki-67 expression level, and the presence of MetS were independently associated with a poorer prognosis for patients with DLBCL based on both PFS and OS. Additionally, the application of rituximab was also identified as an independent factor influencing the prognosis (Table 5).

**Table 5:** Multivariate analysis of clinical characteristics and prognosis in DLBCL

Characteristics	PFS		OS	
	*P*	HR (95% CI)	*P*	HR (95% CI)
IPI	0.042*	0.505 (0.262–0.976)	0.027*	0.355 (0.141–0.890)
Tumor staging	0.701	0.891 (0.494–1.606)	0.851	0.922 (0.395–2.150)
MetS	0.026*	0.624 (0.411–0.946)	0.033*	0.537 (0.304–0.950)
Ki-67	0.025*	0.594 (0.377–0.937)	0.019*	0.443 (0.224–0.874)
LDH	0.366	0.812 (0.518–1.274)	0.518	0.818 (0.445–1.504)
Rituximab	0.013*	1.842 (1.140–2.976)	0.047*	1.902 (1.010–3.583)

*IPI* International prognostic index, *MetS* metabolic syndrome, *LDH* lactate dehydrogenase

*P < 0.05

### Survival analysis

**Fig. 1:**
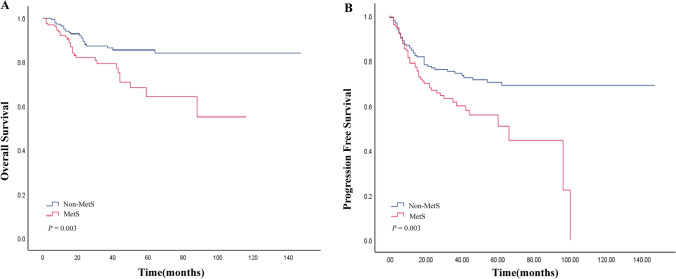
Kaplan–Meier analysis of OS **A** and PFS **B** according to number of MetS components

Patients with pre-treatment combined MetS had a higher mortality rate of 23.3% compared to those without MetS (14.5%). Similarly, the recurrence rate among patients with pre-treatment combined MetS was significantly higher at 39.5% compared to 25.8% for patients without MetS. Additionally, the overall median survival time of patients with pre-treatment combined MetS was lower than that of DLBCL patients. These findings demonstrated a correlation between poorer OS (*P* = 0.003) (Fig. 1a) and PFS (*P* = 0.003) (Fig. 1b) among patients with pre-treatment combined MetS.

## Discussion

Diffuse large B-cell lymphoma is a highly aggressive and heterogeneous type of NHL. The introduction of rituximab has led to a significant improvement in the OS of DLBCL patients, resulting in a cure rate of over 60%, although some patients still experience disease progression [10]. Hence, the focus of our clinic should be directed toward enhancing the prognosis of DLBCL patients and identifying the factors that contribute to disease progression. These issues remain crucial and require further attention in clinical practice.

Metabolic syndrome encompasses a cluster of pathological states, such as obesity (particularly abdominal obesity), a history of diabetes mellitus or elevated fasting glucose, dyslipidemia, and hypertension. The precise pathogenesis of MetS remains elusive, although it is widely believed to arise from a complex interplay of environmental, genetic, and immunological factors. Some research has proposed that insulin resistance serves as the central mechanism of MetS. Insulin resistance weakens the glucose-lowering effect of insulin in the body, leading to compensatory hyperinsulinemia. Moreover, insulin binding to the insulin-like growth factor receptor (IGF-1) on the cell surface promotes cell proliferation and subsequently engenders the development of various malignant tumors [11]. A multitude of studies have highlighted the association between MetS and a wide range of cancer prognoses. For instance, Zhou et al. conducted a study indicating that MetS was correlated with unfavorable outcomes in breast cancer patients undergoing neoadjuvant chemotherapy. This included lower remission rates, heightened risks of recurrence, and increased mortality rates [12]. Further supporting this connection, Li et al. found that heightened expression of the MetS core gene IL6 facilitated the malignant progression of colorectal cancer through the mTOR-S6K signaling pathway [13]. Additionally, Zhang et al. discovered a link between MetS and the heightened pathological stage of renal clear cell carcinoma [14], while research by Sooim Sin et al. identified an increased susceptibility to lung cancer in men with MetS, with a positive correlation between the severity of MetS and the risk of developing cancer [15]. Moreover, epidemiological reports have revealed that survivors of childhood cancers, such as acute lymphoblastic leukemia (ALL), central nervous system tumors, and lymphomas, are more likely to exhibit clinical features of MetS and an increased risk for cardiovascular diseases (CVD) [16].

In this investigation, a retrospective study was carried out to explore the prognostic impact of MetS on DLBCL and its association with clinicopathological features by analyzing 319 patients initially diagnosed with DLBCL at the Affiliated Cancer Hospital of Harbin Medical University. The univariate analysis revealed a significant correlation between MetS and patients’ age, IPI score, the application of rituximab, and Ki-67 expression levels (Table 2). Notably, patients with pre-treatment comorbid MetS were found to be predominantly older, with 66 patients aged 60 years or older, representing over 50% of the cohort. Epidemiologically, the global incidence of metabolic syndrome and cancer is increasing, with a relatively high number of DLBCL patients having an onset age above 60 years, and some even presenting with an onset age above 70 years. Given that elderly individuals are more prone to comorbidities, such as respiratory and circulatory system diseases, as well as metabolic disorders including blood glucose, blood pressure, and lipids, they are at an increased risk of experiencing a poorer prognosis in the context of DLBCL [1]. This connection emphasizes the potential impact of MetS on the clinical outcomes of DLBCL patients within an aging population. In addition, there is a correlation between MetS status groupings and higher IPI scores in patients, encompassing patient age, ECOG, tumor stage, number of extranodal organ involvement, and LDH. Subgroup comparisons revealed that the proportion of patients with pre-treatment MetS who were aged 60 years or older (51.2%) was higher than the proportion of patients without pre-treatment MetS who were aged 38.4%. Furthermore, the proportion of patients with pre-treatment MetS who had tumor stage III–IV (35.7%) was higher than the proportion of patients with pre-treatment MetS who had tumor stage I–II (33.2%). Additionally, the proportion of patients with ECOG ≥ 2 in patients with MetS before treatment (23.3%) was higher than the proportion of patients with ECOG < 2 in patients without MetS before treatment (15.8%), and the proportion of patients with higher than normal LDH levels in patients with MetS before treatment (38%) were higher than the proportion of patients with normal LDH levels in patients with MetS before treatment (20.8%). The significant difference between age and ECOG levels indicates that older patients are more likely to have a poorer tolerance for malignant diseases, such as tumors. Hence, the correlation between IPI scores and MetS status subgroups is complex and may be attributed to the predominant influence of the patient’s age, which cannot be overlooked despite the role of lactate dehydrogenase. Notably, pyruvate produced during glycolysis is converted to lactate, the end product of glycolysis, by lactate dehydrogenase A (LDH-A), an enzyme that recycles the oxidized form of nicotinamide adenine dinucleotide (NAD +) to maintain cellular energy supply. Given these complexities, further scientific studies are warranted to provide supporting evidence [17]. In this study, it was observed that there exists a correlation between the use of rituximab and MetS. Notably, rituximab was relatively new to the domestic healthcare market and initially carried a high cost, making it accessible only to individuals with a certain level of economic means. Consequently, families with higher economic status tended to have a more affluent lifestyle, characterized by a diverse and richer diet, which in turn increased the likelihood of metabolic dysfunction, such as hyperlipidemia, hyperglycemia, and related underlying diseases. Ki-67 was initially identified as an antigen in the nucleus of Hodgkin’s lymphoma cells, and it is highly expressed in circulating cells but strongly down-regulated in quiescent G0 cells. Its expression level reflects the degree of active cell proliferation and has become a clinically important proliferation marker for grading many cancers [18]. Notably, Ki-67 expression levels greater than 70% were found in up to 72.9% of patients with pre-treatment combined MetS. This finding may be related to the highly proliferative and energy-consuming metabolic state of tumor cells. There is a lack of direct correlation reported between metabolic syndrome and Ki-67, which deserves further exploration in subsequent studies. Tumor cells usually need to take up more glucose to produce biomass and support their reimbursed growth. It has now been shown that hyperinsulinemia may promote cancer cell growth through the PI3K/AKT/mTORC and MAPK/ERK signaling cascades [19].

In patients with DLBCL, Kaplan–Meier survival analysis curves indicated a significant association between MetS and a poor prognosis, as well as a heightened risk of recurrence (*P* < 0.05) (Fig. 1). Specifically, the median survival time for patients with pre-treatment comorbid MetS was significantly shorter at 28 months, compared to 37 months for those without pre-treatment comorbid MetS. Furthermore, the median PFS time for patients with pre-treatment comorbid MetS was also notably shorter at 22 months, compared to 30 months for patients without pre-treatment comorbid MetS. Additionally, our investigation delved into the correlation between individual components of MetS and related lipid markers vis-à-vis prognosis, highlighting MetS as being more significantly associated with prognosis than its individual components. The correlation with individual components of the metabolic syndrome has been documented in studies pertaining to lymphoma. Notably, diabetes mellitus has been identified as an independent risk factor for hematological malignancies, with specific causes of death and an increased risk of NHL [20]. However, it is worth noting that the present study did not demonstrate a correlation between hyperglycemia and DLBCL, prompting consideration for subsequent validation through sample enlargement. Moreover, a retrospective study by Suheyla et al. found that BMI did not significantly impact treatment response in DLBCL patients, aligning with the findings of our study [21]. In another retrospective study, lipid levels were observed to elevate in all DLBCL patients following chemotherapy, and apolipoprotein A-I emerged as an independent prognostic factor [22]. These findings provide impetus for further comprehensive investigations into the lipid-DLBCL correlation.

The prognosis of patients with DLBCL has been extensively studied through unifactorial and multifactorial analyses, which have identified several factors associated with prognosis. Specifically, age, Ki-67 expression level, IPI score, LDH expression level, and the application of rituximab were found to have prognostic significance (Tables 3 and 4). Notably, high Ki-67 positivity has been correlated with higher prognostic indices, while Ki-67-negative patients demonstrated higher overall and disease-free survival rates, indicating the potential utility of Ki-67 as a predictive prognostic indicator for DLBCL [23]. Furthermore, multifactorial Cox regression analyses were conducted to investigate the solitary effect of MetS on subsequent survival and disease progression in DLBCL patients. These analyses revealed that the presence of combined MetS, along with IPI scores, Ki-67 expression levels, and the application of rituximab, could serve as independent predictors of prognosis in DLBCL (Table 5).

This study revealed, for the first time, a correlation between the presence of metabolic syndrome (MetS) in DLBCL patients and several crucial factors, namely age, IPI score, use of rituximab, and levels of Ki-67 expression. Additionally, it was evident that patients with MetS experienced a poorer prognosis. This implies that MetS, along with IPI score, Ki-67 expression levels, and the use of rituximab, could serve as independent risk factors affecting the prognosis of DLBCL patients.

This study has certain limitations. Firstly, the small sample size included in the study hindered the assessment of lipid-related indexes in DLBCL patients at the time of initial treatment. Many DLBCL patients lacked complete and detailed lipid-related clinical data before treatment, which also made it impossible to assess whether the patients were combined with MetS before treatment. To ensure the authenticity and completeness of the clinical data, DLBCL patients were screened in strict accordance with the exclusion and exclusion indexes, thereby leading to the exclusion of a substantial number of cases with a primary diagnosis of DLBCL. Additionally, DLBCL patients with combined MetS were not followed up in this study to assess their use of hypoglycemic, antihypertensive, or lipid-lowering medications during treatment, and whether the choice and application of medications impacted the time patients received maintenance therapy and the prognostic impact of MetS. Mafiana et al. [24] found that colorectal cancer patients with MetS who were not treated with metformin showed improved overall patient survival when taking metformin and statins during treatment. Another study [25] demonstrated that the use of metformin and statins reduced the risk of hepatocellular carcinoma development and improved MetS symptoms. These findings suggest that pharmacological treatment to improve the MetS status can have a mitigating effect on the poor prognosis of tumor patients.

## Conclusion

In conclusion, the study provides an inaugural exploration of the link between MetS and prognosis in patients with DLBCL by utilizing available clinical data. It validates the need for clinical guidelines for the care of pre-treatment patients with MetS and confirms MetS as an independent influencing factor for the prognosis of DLBCL patients. Furthermore, the results highlight how crucial it is to raise public awareness. To clarify the role of MetS in the onset and prognosis of DLBCL and open up new directions for clinical diagnosis and treatment targets, more prospective studies are necessary.

## Data Availability

Not applicable.

## Abbreviations

DLBCL: Diffuse large B-cell lymphoma
OS: Overall survival
PFS: Progression-free survival
NHL: Non-Hodgkin’s lymphoma
MetS: Metabolic syndrome
GCB: Germinal center B-cell
non-GCB: Non-germinal center B-cell
IPI: International prognostic index
ECOG: Eastern cooperative oncology group
LDH: Lactate dehydrogenase
β2-MG: β2-Microglobulin
NCEP-ATP III: National Cholesterol Education Program Adult Treatment Panel III
BMI: Body mass index
TG: Triglycerides
HDL-C: High-density lipoprotein cholesterol
LDL-C: Low-density lipoprotein cholesterol
SBP: Systolic blood pressure
DBP: Diastolic blood pressure
FPG: Fasting plasma glucose
TC: Total cholesterol
IGF-1: Insulin-like growth factor receptor
ALL: Lymphoblastic leukemia
CVD: Cardiovascular diseases
LDH-A: Lactate dehydrogenase A
NDA+: Nicotinamide adenine dinucleotide
